# On the Use of Turpentine in Puerperal Fever

**Published:** 1825-07

**Authors:** J. Kinneir


### Art. VII.—*On the Use of Turpentine in Puerperal Fever.* By J. Kinneir, M.D.

In no disease, perhaps, has a greater contrariety of sentiment, both with respect to the pathology and treatment, obtained among medical men, than in puerperal fever. This is much to be regretted, as there is probably no disease which, at its onset, requires a more prompt and energetic practice, or which, if left to the unaided efforts of nature, proves more certainly destructive to life. The late publications of Drs. Armstrong, Douglas, Campbell, and Mackintosh, have, however, thrown much light on the disease, and established its pathology on a tolerably firm basis. It is now pretty generally admitted that abdominal inflammation constitutes the source of danger.

In Dr. Campbell's Treatise, I find much to commend; but, on the other hand, there are passages which seem to me to merit reprehension; and, though it may be thought rather invidious to comment on a contemporary's practice, I trust I shall, in the present instance, be held excusable, as the following remarks are dictated solely by a regard for the interests of humanity. Much credit, I conceive, is due to Dr. Campbell for the ardour with which he has prosecuted his pathological inquiries, and for his bold and energetic treatment with respect to blood-letting. He does not seem, however, to have been very judicious in his selection of purgatives, as the only ones he enumerates in his cases are ol. ricini, and ℥jss. and ℥ij. doses of sodæ sulphat. and magnesiæ sulphat. What peculiar advantages could result from giving a patient two ounces of sulphate of soda, I am totally unable to conceive; and where nausea is present, as is generally the case in this disease, so large a dose would, I apprehend, be very likely to aggravate that distressing symptom. Dr. Armstrong's practice of giving a scruple of submuriate of mercury, followed by a dose of a purgative mixture every hour, till the bowels had been freely evacuated, I have found to be by far the best mode of giving cathartics. Dr. C., indeed, frequently ordered two or three-grain doses of calomel; but, as they were always combined with pulv. antimonialis, they seem to have been ordered rather with the view of determining to the surface, and equalising the circulation, than of producing a cathartic operation.

It is not, however, the kind of purgative which Dr. C. prescribes, the doses, or mode of administering them, which I consider so reprehensible, as his ridicule of a remedy which, in my opinion, and that of some of my friends, is by far the most valuable one which has been hitherto used in this complaint:—I mean the oleum terebinthinæ. The silent indifference with which Dr. C. treated the virtues of this remedy, in his first observations on Puerperal Fever, though recommended by high authority, and his subsequent ridicule of the practice, which (according to his own statement) he had never tried, are, I conceive, by no means creditable to him as a medical inquirer; particularly as his own cases sufficiently evince that his treatment was by no means very successful. In some of his cases, which terminated fatally, if, instead of wine and spirits, and the fol. calid. abdomini, he had ordered the abdomen to be steeped with tepid oil of turpentine, and had recourse to its internal use, in the doses which I shall presently recommend, unless the πειρα σφαλερη be peculiarly applicable to me, the result would have been very different. Dr. C. is lavish of his ridicule on Dr. Payne, because, in a "printed book," he thought proper to recommend the ol. terebinthinæ in this disease; but the witticism respecting the *lapis besoardicus*, burnt cork, living slaters, &c. is, I conceive, entirely pointless, as there is not the slightest analogy, in physical properties, between any of these articles and the rectified oil of turpentine. Dr. C. is incorrect in stating that the exhibition of oil of turpentine in this complaint, has been found useful in only a few instances. Besides, Drs. Brennan and Douglas, and several respectable practitioners, have recorded cases, in the different periodical publications, illustrative of its singular efficacy. In the Dublin Hospital Reports for 1822, Dr. Douglas, an eminent and experienced accoucheur, in a tract on Puerperal Fever, makes the following remarks:—"I would not feel as if I were doing justice to the community, if I did not distinctly state that I consider it, (oil of turpentine,) when judiciously administered, more generally suitable, and more effectually remedial, than any other medicine yet proposed. I can safely aver I have seen women recover, apparently by its influence, from almost hopeless conditions: certainly after every hope of recovery, under ordinary treatment, had been relinquished."

About four years ago, several cases of puerperal fever occurred in the practice of an eminent surgeon in Northamptonshire. In the first two or three instances, though the usual remedies, (venesection, purging, &c.) were had recourse to, with promptitude and decision, the termination was fatal. This circumstance induced him to give a trial to the ol. terebinthinæ, as recommended by Brennan, and with the most happy effects, as all the cases in which he exhibited it terminated favourably. From witnessing its effect in these cases, this gentleman, who is a man of great experience and observation, was so fully impressed with a conviction of its efficacy in puerperal fever, that he has since declared to me, in conversation, he considered the practice introduced by Brennan to be one of the most important discoveries in modern medicine.

Candour induces me to remark, that the puerperal epidemic which prevailed in Dublin and some other places, in which the rectified oil of turpentine was found so eminently useful, seems to have been of a somewhat different type from that which Dr. C. and his professional brethren witnessed in Edinburgh: that, in the latter, the complaint seems to have exhibited symptoms of greater excitement, and consequently required a treatment in some measure diversified. Still, however, I conceive that a similar practice, with some modifications, according as the symptoms are mild or severe, will be found most successful in all cases of puerperal fever. For it has fallen to my lot to witness, within the last ten years, several cases of the disease, some occurring epidemically, and others sporadically, in different localities; and in all a similar plan of treatment was adopted, with the happiest results. When the symptoms were urgent, the copious abstraction of blood at the onset, so as to produce a decisive effect, active cathartics, and the internal and external use of rectified oil of turpentine, were resorted to, and succeeded in more than nine cases out of ten. In the milder cases, venesection was sometimes dispensed with, and active purgation, with the external application of tepid oleum terebinthinæ to the abdomen, were alone adequate to the cure.

Some practitioners, impressed with an idea of the peculiar and mysterious nature of puerperal fever, will perhaps object, that peritonitis, occurring after parturition, has been often mistaken for it; but I confess that I am unable to draw a line of demarcation, if any such do exist, between the two diseases; and this, I conceive, is of the less importance, as they require a similar practice. When I found, occurring a few days after parturition, an acute fixed pain in the abdomen, aggravated on pressure, or a general soreness of the abdomen, rendered more acute by pressure, a wildness of the eyes, hurried inspiration, quick pulse, and much uneasiness on turning to either side, I never hesitated to term such a combination of symptoms puerperal fever.

The mode of administering the ol. terebinthinæ in one-ounce doses, as tried by Professor Hamilton and others, is, in my opinion, an injudicious one. From one to two drachms of the rectified oil in mist. amygdal, or with an equal quantity of syrup and an ounce of distilled water, in the form of a draught, should be exhibited after bleeding and purging, every three or four hours, until a considerable abatement of the pain and other symptoms take place. It will be rarely requisite to repeat the draught more than two or three times. Should the symptoms return, the medicine may again be resorted to. In this manner the remedy has been frequently prescribed by myself and others, with the happiest results. The above draught, in general, has seemed to relax the intestines, or, at least, to sustain the cathartic operation produced by preceding remedies. It has been but rarely rejected by the stomach. In every instance of puerperal fever, fomenting the abdomen with tepid oil of turpentine, is (I think) adviseable, as I have frequently known females express, in the most forcible terms, the extraordinary and almost instantaneous diminution of pain which they have experienced from its application.

Many practitioners are (I believe) deterred from using, in puerperal fever, the ol. terebinthinæ, from an apprehension of its proving injurious in a disease so highly inflammatory; but the nature of inflammation, and the agency of some remedies in its removal are still involved in great obscurity. Facts, however, are "stubborn things," and should not be overlooked or disregarded by the medical practitioner, from his inability to explain them, or because they may not tally with his preconceived notions of disease. In burns and scalds, which may be regarded as external phlegmasiæ, no experienced surgeon in the present day questions the singular efficacy of spirits of turpentine, externally applied, in the manner recommended by Kentish and others; and in scarlatina and rubeola, diseases of high excitement, who would, *a priori*, expect benefit from the internal exhibition of carbonate of ammonia?

Though I do not consider the ol. terebinthinæ in puerperal fever, to be a specific, in the sense usually attached to the term, or think that, in the early stage of the majority of instances, it ought to supersede the fearless use of the lancet, I am perfectly convinced that it is, when judiciously administered, a medicine of singular efficacy in this disease; and that it will not unfrequently succeed, after the failure of the usual remedies, in rescuing patients from a state apparently hopeless.

***Provost-street, City-road; May* 1825.**

